# Modeling Tensile Damage and Fracture Behavior of Fiber-Reinforced Ceramic-Matrix Minicomposites

**DOI:** 10.3390/ma13194313

**Published:** 2020-09-27

**Authors:** Zhongwei Zhang, Yufeng Liu, Longbiao Li, Daining Fang

**Affiliations:** 1 Institute of Advanced Structure Technology, Beijing Institute of Technology, Beijing 100081, China; fangdn@pku.edu.cn; 2 Science and Technology of Advanced Functional Composite Materials Laboratory, Aerospace Research Institute of Materials & Processing Technology, Beijing 100076, China; liuyf123@csu.edu.cn; 3 Powder Metallurgy Research Institute, Central South University, Changsha 410083, China; 4 College of Civil Aviation, Nanjing University of Aeronautics and Astronautics, No.29, Yudao St., Nanjing 210016, China

**Keywords:** ceramic-matrix composites (CMCs), minicomposite, tensile, damage, fracture

## Abstract

Evolution of damage and fracture behavior of fiber-reinforced mini ceramic-matrix composites (mini-CMCs) under tensile load are related to internal multiple damage mechanisms, i.e., fragmentation of the brittle matrix, crack defection, and fibers fracture and pullout. In this paper, considering multiple micro internal damage mechanisms and related models, a micromechanical constitutive stress–strain relationship model is developed to predict the nonlinear mechanical behavior of mini-CMCs under tensile load corresponding to different damage domains. Relationships between multiple micro internal damage mechanisms mentioned above and tensile micromechanical multiple damage parameters are established. Experimental tensile nonlinear behavior, internal damage evolution, and micromechanical tensile damage parameters corresponding to different damage domains of two different types of mini-CMCs are predicted. The effects of constitutive properties and damage-related parameters on nonlinear behavior of mini-CMCs are discussed.

## 1. Introduction

With the development of thermodynamics, the advancement of component integrated design technology, the weight reduction brought by structural simplification and the comprehensive development of material technology, the thrust-to-weight ratio of aeroengines has gradually increased. However, on the premise of maintaining the engine layout and not changing the conventional metal materials, the improvement of aerodynamic, thermal, component design and structural weight reduction techniques can only increase the ratio between the thrust and weight of the aeroengines to approximately 14. For aeroengines with ratios between the thrust and a weight of approximately 12–15 or higher, more breakthroughs must be made in new materials, new process applications and new structural design, such as polymer-matrix composites (PMCs) or metal-matrix composites (MMCs) for cold section components in aeroengine and ceramic-matrix composites (CMCs) for hot section components in aeroengines. Two main types of CMCs are used in aeroengines, including, C/SiC and SiC/SiC. For aeroengines, the operating temperatures of C/SiC and SiC/SiC are approximately 1650 °C and 1450 °C, respectively. Increasing the operating temperature of SiC fibers can raise the operating temperature of SiC/SiC to 1650 °C. Results show that the application of CMCs in the hot section components of the combustion chamber, turbine, and nozzle can increase the operating temperature of the aeroengine by 300–500 °C, reduce the weight by 50–70% and increase the thrust by 30–100% [1,2,3,4].

In order to safely and confidently implement CMCs in different thermo-structural component applications, one should understand the complexity of designing with such brittle composite systems. The mechanical behavior of minicomposites can be used to analyze the internal complex damage evolution of woven composites [5,6,7,8]. Many in situ monitoring techniques, including acoustic emission (AE), X-ray microtomography, and acousto-ultrasonics (AU), have been used to detect and reveal internal damage evolution mechanisms in minicomposites [9,10,11,12]. Under tensile load, the damages in the minicomposites can be divided into different stages and the damage stages are affected by the fiber type and interphases [13,14,15,16]. Heat-treatment at elevated temperature causes damages in the fiber and interphase, leading to the degradation of tensile performance of mini-CMCs [17,18,19,20,21].

The objective in the present analysis is to build the relationship between tensile nonlinear damage behavior and multiple micro internal damage mechanisms of mini-CMCs. A micromechanical nonlinear stress–strain relationship model is given to predict the tensile stress–strain relationship of mini-CMCs when either partial or complete interfacial debonding occurs. Experimental tensile damage evolution and related multiple microdamage parameters of two different mini-CMCs are predicted. Effects of constitutive properties and damage-related parameters on damage and fracture of mini-CMCs are analyzed.

## 2. Theoretical Analysis

The tensile nonlinear behavior of mini-CMCs is mainly attributed to multiple microdamage mechanisms. Through in situ experimental observations and monitoring on internal damage state [12,13], damage evolution and fracture of mini-CMCs under tensile load can be divided into three main domains, including:

(1) Domain I, the linear domain.
(2) Domain II, the nonlinear domain due to microdamages of matrix fragmentation.
(3) Domain III, the secondary linear and final fracture domain due to gradual fibers fracture and pullout.

In this section, the micromechanical nonlinear constitutive relationship of three damage domains mentioned above is developed and related to the microdamage state of matrix fragmentation and fiber fractures and pullouts inside of mini-CMCs. A micromechanical parameter of interface debonding fraction *η* is adopted to describe composite internal damage evolution.

### 2.1. Domain I

In Domain I, no damages occur in mini-CMCs, and the linear relationship between applied stress *σ* and composite stress–strain ε_c_ can be determined by Equation (1) [4].

(1) $$\varepsilon_{c}=\frac{\sigma }{E_{c}}$$

where *E*_c_ is the elastic modulus of the undamaged composite.

### 2.2. Domain II

When multiple microdamage mechanisms occur in mini-CMCs, the micro stress field of the damaged composite is affected by composite constitutive properties (i.e., volume of the fiber and matrix *V*_f_ and *V*_m_, and fiber radius *r*_f_) and damage state (i.e., fragmentation length of the brittle matrix *l*_c_, and crack defection length at the interface *l*_d_). The fiber axial stress distribution can be determined by Equation (2) [4].

(2) $$\sigma_{f}\left(x\right)=\left\{ \begin{array}{l} \frac{\sigma }{V_{f}}-\frac{2\tau_{i}}{r_{f}}x,x\in \left[0,l_{d}\right]\\ \sigma_{\mathrm{fo}}+\left(\frac{V_{m}}{V_{f}}\sigma_{\mathrm{mo}}-2\frac{l_{d}}{r_{f}}\tau_{i}\right)\exp\left(-\rho \frac{x-l_{d}}{r_{f}}\right),x\in \left[l_{d},\frac{l_{c}}{2}\right] \end{array}\right.$$

where *τ*_i_ the interface frictional shear stress, σ_fo_ and σ_mo_ the axial stress of the fiber and matrix in the interface bonding region, and ρ the shear–lag model parameter. The stochastic model in Equation (3) can be used to determine the fragmentation length of the brittle matrix [22], and the relationship between the applied stress and debonding length at the interface is obtained by Equation (4) [23].

(3) $$l_{c}=l_{\mathrm{sat}}{\left\{1-\exp\left[-{\left(\frac{\sigma_{m}}{\sigma_{R}}\right)}^{m}\right]\right\}}^{-1}$$

(4) $$l_{d}=\frac{r_{f}}{2}\left(\frac{V_{m}E_{m}\sigma }{V_{f}E_{c}\tau_{i}}-\frac{1}{\rho }\right)-\sqrt{{\left(\frac{r_{f}}{2\rho }\right)}^{2}+\frac{r_{f}V_{m}E_{m}E_{f}}{E_{c}\tau_{i}^{2}}\zeta_{d}}$$

where *l*_sat_ is the saturation matrix fragmentation length, *σ*_m_ is the stress carried by the matrix, *σ*_R_ is the characteristic stress for matrix fragmentation, and ζ_d_ is the debonding energy of the interface.

The composite strain ε_c_ is equal to the strain of intact fibers, and the nonlinear stress–strain constitutive relationship can be determined by Equation (5).

(5) $$\varepsilon_{c}\left(\sigma \right)=\left\{ \begin{array}{l} \frac{\sigma }{V_{f}E_{f}}\eta -\frac{\tau_{i}}{E_{f}}\frac{l_{d}}{r_{f}}\eta +\frac{\sigma_{\mathrm{fo}}}{E_{f}}\left(1-\eta \right)-\frac{1}{\rho E_{f}}\frac{2r_{f}}{l_{c}}\left(\frac{V_{m}}{V_{f}}\sigma_{\mathrm{mo}}-2\frac{l_{d}}{r_{f}}\tau_{i}\right)\\ \;\;\;\times \left[\exp\left(-\frac{\rho }{2}\frac{l_{c}}{r_{f}}\left(1-\eta \right)\right)-1\right]-\left(\alpha_{c}-\alpha_{f}\right)\Delta Τ,\eta <1\\ \frac{\sigma }{V_{f}E_{f}}-\frac{\tau_{i}}{2E_{f}}\frac{l_{c}}{r_{f}}-\left(\alpha_{c}-\alpha_{f}\right)\Delta Τ,\eta =1 \end{array}\right.$$

where *E*_f_ is the elastic modulus of the fiber, *η* is the ratio between debonding length *l*_d_ and half of fragmentation length of the brittle matrix *l*_c_, α_f_ and α_c_ are the axial thermal expansion coefficient of the fiber and the composite, and ΔT is the temperature difference between testing and fabricated temperature.

### 2.3. Domain III

Upon approaching saturation of fragmentation of the brittle matrix, some fibers gradually fracture and pullout inside of the minicomposite. Considering intact and broken fiber in the different damage regions, the axial stress of the fiber can be determined by Equation (6) [4].

(6) $$\sigma_{f}\left(x\right)=\left\{ \begin{array}{l} \Phi -\frac{2\tau_{i}}{r_{f}}x,x\in \left[0,l_{d}\right]\\ \sigma_{\mathrm{fo}}+\left(\Phi -\sigma_{\mathrm{fo}}-2\frac{l_{d}}{r_{f}}\tau_{i}\right)\exp\left(-\rho \frac{x-l_{d}}{r_{f}}\right),x\in \left[l_{d},\frac{l_{c}}{2}\right] \end{array}\right.$$

where Φ is the stress of unbroken fiber, which can be determined by Equation (7) [24].

(7) $$\frac{\sigma }{V_{f}}=\Phi \left(1-P\right)+\frac{2\tau_{i}}{r_{f}}\langle L\rangle P$$

where 〈L〉 is the average pullout length of a broken fiber, and *P* the probability of fiber being broken.

Considering fibers fracture inside of mini-CMCs, the nonlinear stress–strain relationship at damage Domain III is acquired by Equation (5).

(8) $$\varepsilon_{c}\left(\sigma \right)=\left\{ \begin{array}{l} \frac{\Phi }{E_{f}}\eta -\frac{\tau_{i}}{E_{f}}\frac{l_{d}}{r_{f}}\eta +\frac{\sigma_{\mathrm{fo}}}{E_{f}}\left(1-\eta \right)-\frac{1}{\rho E_{f}}\frac{2r_{f}}{l_{c}}\left(\Phi -\sigma_{\mathrm{fo}}-2\frac{l_{d}}{r_{f}}\tau_{i}\right)\\ \;\;\;\times \left[\exp\left(-\frac{\rho }{2}\frac{l_{c}}{r_{f}}\left(1-\eta \right)\right)-1\right]-\left(\alpha_{c}-\alpha_{f}\right)\Delta Τ,\eta <1\\ \frac{\Phi }{E_{f}}-\frac{\tau_{i}}{E_{f}}\frac{l_{c}}{2r_{f}}-\left(\alpha_{c}-\alpha_{f}\right)\Delta Τ,\eta =1 \end{array}\right.$$

## 3. Experimental Comparison

Nonlinear tensile damage and fracture behavior of two different types of mini-CMCs are predicted. The effect of heat treatment at 1300 and 1500 °C on tensile nonlinear damage development behavior of mini-CMCs at room temperature is also analyzed. The material properties of different mini-CMCs are listed in Table 1.

### 3.1. SiC/SiC Minicomposite

**Figure 1:**
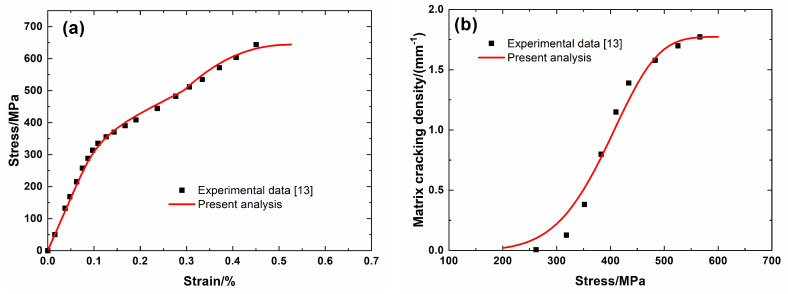
(**a**) Tensile nonlinear stress–strain curves; and, (**b**) matrix fragmentation density versus applied stress curves of Hi-Nicalon^TM^ SiC/SiC mini ceramic-matrix composites (mini-CMCs).

Figure 1 shows the experimental data and theoretical predicted results of tensile nonlinear curves and fragmentation density of brittle matrix versus applied stress curves of Hi-Nicalon^TM^ SiC/SiC mini-CMC. The predicted results of tensile curves and fragmentation density of brittle matrix evolution curves both agreed well with experimental data. Three main damage domains are obtained from the nonlinear tensile curve, including:

(1) Domain I, the linear domain. The domain starts from the initial loading to the first matrix fragmentation stress *σ*_mc_ = 200 MPa, and the first fragmentation density of the matrix is approximately *λ*_mc_ = 0.02/mm, and the corresponding composite tensile strain is approximately *ε*_c_ = 0.06%.
(2) Domain II, the nonlinear region due to matrix fragmentation. The domain starts from *σ*_mc_ = 200 MPa to the saturation matrix fragmentation stress *σ*_sat_ = 560 MPa, and the saturation fragmentation density of the matrix is approximately *λ*_sat_ = 1.76/mm, and the composite tensile strain is approximately *ε*_c_ = 0.34%.
(3) Domain III, the secondary linear and final fracture domain. The domain starts from *σ*_sat_ = 560 MPa to composite tensile strength *σ*_uts_ = 644 MPa.

**Figure 2:**
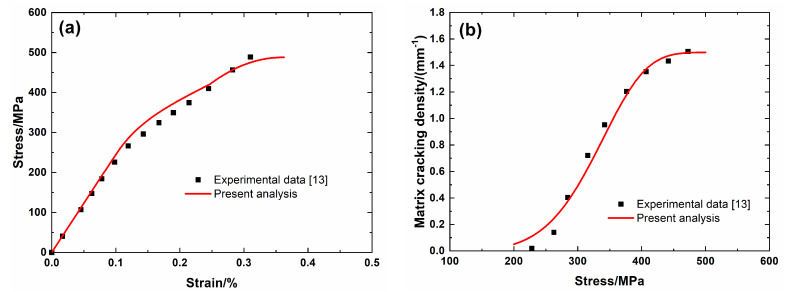
(**a**) Tensile nonlinear stress–strain curves; and, (**b**) matrix fragmentation density versus applied stress curves of Hi-Nicalon^TM^ Type S SiC/SiC mini-CMC.

Figure 2 shows experimental data and theoretical predicted results of tensile nonlinear curves and fragmentation density of the brittle matrix versus applied stress curves of Hi-Nicalon^TM^ Type S SiC/SiC minicomposite. The predicted results of tensile curves and fragmentation density of brittle matrix evolution curves agree with experimental data. Three main damage domains are obtained from the nonlinear tensile curve, including:

(1) Domain I, the linear domain. The domain starts from initial loading to *σ*_mc_ = 200 MPa with *λ*_mc_ = 0.05/mm and *ε*_c_ = 0.08%.
(2) Domain II, the nonlinear region due to matrix fragmentation. The domain starts from *σ*_mc_ = 200 MPa to *σ*_sat_ = 470 MPa with *λ*_sat_ = 1.49/mm and *ε*_c_ = 0.3%.
(3) Domain III, the secondary linear and final fracture region. The domain starts from *σ*_sat_ = 470 MPa to *σ*_uts_ = 488 MPa.

**Figure 3:**
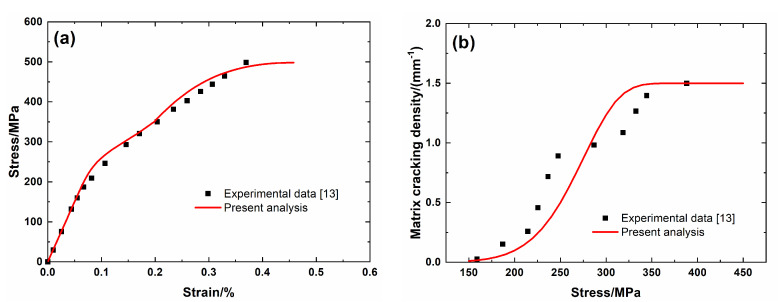
(**a**) Tensile nonlinear stress–strain curves; and, (**b**) matrix fragmentation density versus applied stress curves of Tyranno^TM^ ZMI SiC/SiC mini-CMC.

Figure 3 shows experimental data and theoretical predicted results of tensile nonlinear curves and fragmentation density evolution of brittle matrix curves versus applied stress of Tyranno^TM^ ZMI SiC/SiC minicomposite. Predicted results of tensile curves and fragmentation density of brittle matrix evolution curves agree well with experimental data. Three main damage domains are obtained from the nonlinear tensile curve, including:

(1) Domain I, the linear region. The domain starts from initial loading to *σ*_mc_ = 150 MPa with *λ*_mc_ = 0.01/mm and *ε*_c_ = 0.05%.
(2) Domain II, the nonlinear region due to matrix fragmentation. The domain starts from *σ*_mc_ = 150 MPa to *σ*_sat_ = 350 MPa with *λ*_mc_ = 1.49/mm and *ε*_c_ = 0.198%.
(3) Domain III, the secondary linear and final fracture region. The domain starts from *σ*_sat_ = 350 MPa to *σ*_uts_ = 498 MPa.

**Figure 4:**
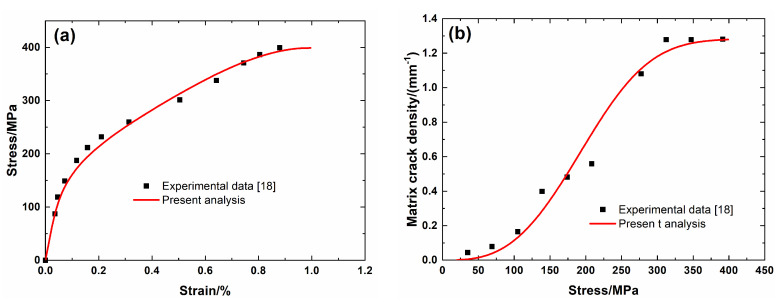
(**a**) Tensile nonlinear stress–strain curves; and, (**b**) matrix fragmentation density versus applied stress curves of SiC/SiC mini-CMC without heat treatment.

Figure 4 shows experimental data and theoretical predicted results of tensile nonlinear curves and fragmentation density evolution of the brittle matrix versus applied stress of SiC/SiC minicomposite without heat treatment. The predicted results of tensile curves and fragmentation density evolution of the brittle matrix curves agree well with experimental data. Three main damage domains are obtained from the nonlinear tensile curve, including:

(1) Domain I, the linear region. The domain starts from initial loading to *σ*_mc_ = 35 MPa with *λ*_mc_ = 0.04/mm and *ε*_c_ = 0.013%.
(2) Domain II, the nonlinear region. The domain starts from the *σ*_mc_ = 35 MPa to *σ*_sat_ = 340 MPa with *λ*_sat_ = 1.27/mm and *ε*_c_ = 0.6%.
(3) Domain III, the secondary linear and final fracture domain. The domain starts from *σ*_sat_ = 340 MPa to *σ*_uts_ = 399 MPa.

**Figure 5:**
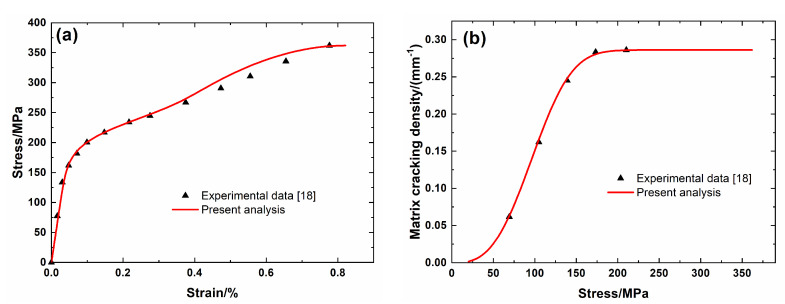
(**a**) Tensile nonlinear stress–strain curves; and, (**b**) matrix fragmentation density versus applied stress curves of SiC/SiC mini-CMC after heat treatment at 1300 °C in Ar atmosphere.

Figure 5 shows experimental data and theoretical predicted results of tensile nonlinear curves and fragmentation density evolution of brittle matrix versus applied stress of SiC/SiC minicomposite after heat treatment at 1300 °C. The predicted results of tensile curves and fragmentation density evolution of brittle matrix versus applied stress curves agree well with experimental data. Three main damage domains are obtained from the nonlinear tensile curve, including:

(1) Domain I, the linear region. The domain starts from initial loading to *σ*_mc_ = 50 MPa with *λ*_mc_ = 0.02/mm and *ε*_c_ = 0.012%.
(2) Domain II, the nonlinear region due to matrix fragmentation. The domain starts from *σ*_mc_ = 50 MPa to *σ*_sat_ = 210 MPa with *λ*_sat_ = 0.28/mm and *ε*_c_ = 0.13%.
(3) Domain III, the secondary linear and final fracture region. The domain starts from *σ*_sat_ = 210 MPa to *σ*_uts_ = 362 MPa.

**Figure 6:**
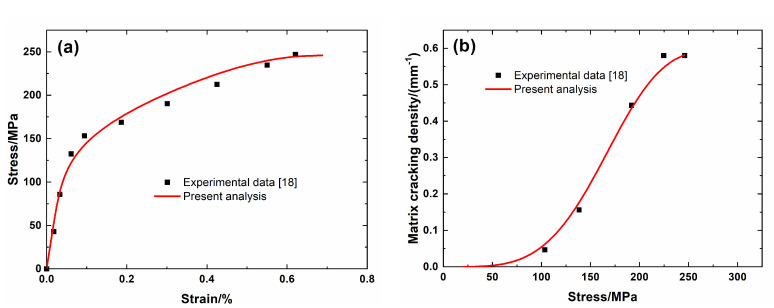
(**a**) Tensile nonlinear stress–strain curves; and, (**b**) matrix fragmentation density versus applied stress curves of SiC/SiC mini-CMC after heat treatment at 1500 °C in Ar atmosphere.

Figure 6 shows experimental data and theoretical predicted results of tensile nonlinear curves and fragmentation density evolution of brittle matrix versus applied stress of SiC/SiC minicomposite after heat treatment at 1500 °C. The predicted results of tensile curves and fragmentation density of brittle matrix versus applied stress curves both agree well with experimental data. Three main damage domains are obtained from the nonlinear tensile curve, including:

(1) Domain I, the linear region. The domain starts from initial loading to *σ*_mc_ = 50 MPa with *λ*_mc_ = 0.005/mm and *ε*_c_ = 0.017%.
(2) Domain II, the nonlinear region. The domain starts from *σ*_mc_ = 50 MPa to *σ*_sat_ = 220 MPa with *λ*_sat_ = 0.58/mm and *ε*_c_ = 0.39%.
(3) Domain III, the secondary linear and final fracture domain. The domain starts from *σ*_sat_ = 220 MPa to *σ*_uts_ = 246 MPa.

### 3.2. C/SiC Minicomposite

**Figure 7:**
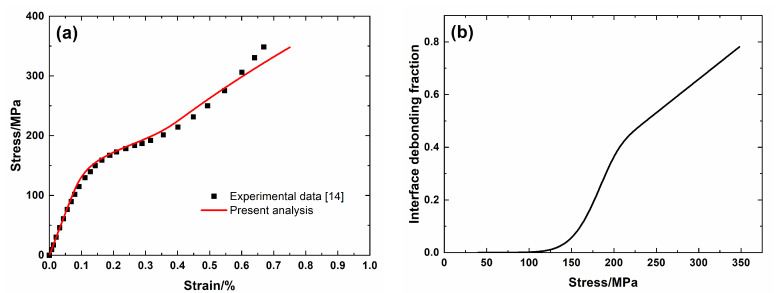
(**a**) Tensile nonlinear stress–strain curves; and, (**b**) the interface debonding fraction versus applied stress curves of unidirectional T–300^TM^ C/SiC mini-CMC.

Figure 7 shows experimental data and theoretical predicted results of tensile curves and the interface debonding fraction versus applied stress curves of unidirectional C/SiC minicomposite. The predicted results of tensile curves agree with experimental data, as shown in Figure 7a, and the composite tensile fractures at the condition of partial interface debonding, as shown in Figure 7b. Three main damage domains are obtained from the nonlinear tensile curve, including:

(1) Domain I, the linear region. The domain starts from initial loading to *σ*_mc_ = 130 MPa.
(2) Domain II, the nonlinear region. The domain starts from *σ*_mc_ = 130 MPa to *σ*_sat_ = 250 MPa.
(3) Domain III, the secondary linear and final fracture domain. The domain starts from *σ*_sat_ = 250 MPa to *σ*_uts_ = 348 MPa. The interface debonding fraction increases to 2*l*_d_/*l*_c_ = 0.78 at tensile strength *σ*_uts_ = 348 MPa.

## 4. Discussion

In this section, the constitutive properties and damage-related parameters on tensile nonlinear stress–strain curves of SiC/SiC mini-CMC are discussed.

### 4.1. Effect of Fiber Volume

**Figure 8:**
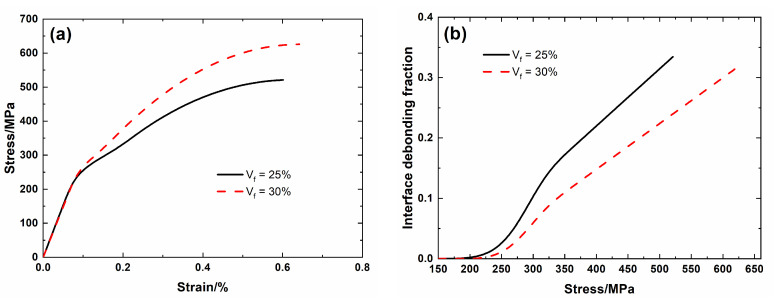
(**a**) Tensile nonlinear stress–strain curves; and, (**b**) the debonding fraction versus applied stress curves of SiC/SiC mini-CMC for different fiber volumes.

Figure 8 shows the tensile nonlinear curves and the evolution of interface debonding fraction for different fiber volumes. For low fiber volume *V*_f_ = 25%, the debonding fraction increases from *η* (*σ*_d_ = 166 MPa) = 0 to *η* (*σ*_uts_ = 521 MPa) = 0.33, and the failure strain of the composite is *ε*_f_ (*σ*_uts_ = 521 MPa) = 0.6%. For high fiber volume *V*_f_ = 30%, the debonding fraction increases from *η* (*σ*_d_ = 204 MPa) = 0 to *η* (*σ*_uts_ = 626 MPa) = 0.32, and the failure strain of the composite is *ε*_f_ = 0.64%. At high fiber volume, the debonding fraction at the same applied stress decreases, and the composite strain at nonlinear Domain II decreases, and the composite tensile strength and failure strain increase.

### 4.2. Effect of Interface Properties

**Figure 9:**
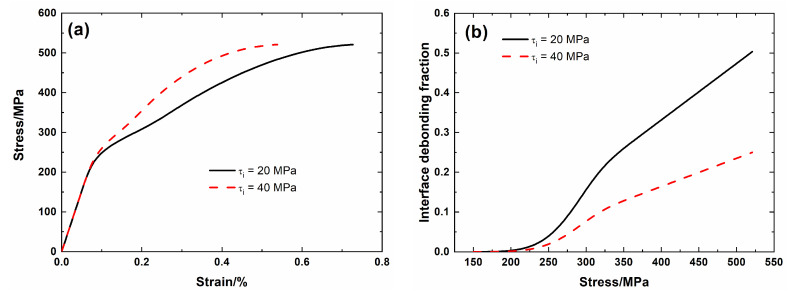
(**a**) Tensile nonlinear stress–strain curves; and, (**b**) the fraction of the interface debonding versus applied stress curves of SiC/SiC mini-CMC for different interface shear stress.

Figure 9 shows the tensile nonlinear curves and the evolution of the interface debonding fraction for different interface shear stresses. For low interface shear stress τ_i_ = 20 MPa, the debonding fraction increases from *η* (*σ*_d_ = 166 MPa) = 0 to *η* (*σ*_uts_ = 521 MPa) = 0.5, and the failure strain of the composite is *ε*_f_ = 0.73%. For high interface shear stress τ_i_ = 40 MPa, the debonding fraction increases from *η* (*σ*_d_ = 168 MPa) = 0 to *η* (*σ*_uts_ = 521 MPa) = 0.25; and the failure strain of the composite is *ε*_f_ = 0.25%. At high interface shear stress, the debonding fraction at the same applied stress decreases, and the composite strain at nonlinear Domain II decreases, and the composite failure strain decreases.

**Figure 10:**
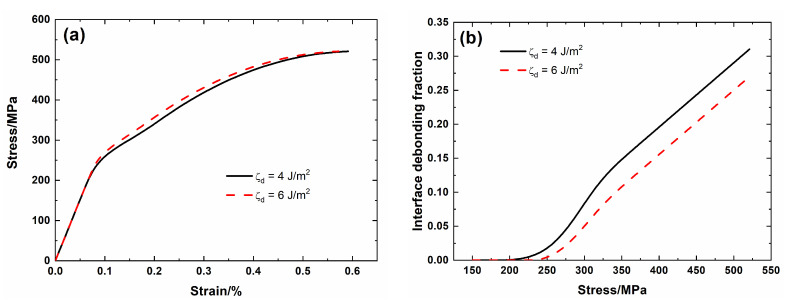
(**a**) Tensile nonlinear stress–strain curves; and, (**b**) the fraction of the interface debonding versus applied stress curves of SiC/SiC mini-CMC for different interface debonding energy.

Figure 10 shows the tensile nonlinear curves and the evolution of the interface debonding fraction for different interface debonding energy. For low interface debonding energy *ζ*_d_ = 4 J/m^2^, the debonding fraction increases from *η* (*σ*_d_ = 192 MPa) = 0 to η (*σ*_uts_ = 521 MPa) = 0.31, and the failure strain of the composite is *ε*_f_ = 0.59%. For high interface debonding energy *ζ*_d_ = 6 J/m^2^, the debonding fraction increases from *η* (*σ*_d_ = 234 MPa) = 0 to *η* (*σ*_uts_ = 521 MPa) = 0.57, and the failure strain of the composite is *ε*_f_ = 0.57%. At high interface debonding energy, the interface debonding stress increases, the debonding fraction at the same applied stress decreases, and the composite strain during nonlinear Domain II decreases, and the failure strain of the composite decreases.

## 5. Conclusions

In this paper, the tensile damage and fracture behavior of two different mini-CMCs are investigated. Multiple microdamage mechanisms of matrix fragmentation, fibers failure and pullout are considered in the analysis of tensile nonlinear curves. Experimental tensile nonlinear curves and internal damage evolution of SiC/SiC and C/SiC mini-CMCs are predicted. The effects of material properties and damage-related parameters on tensile damage and fracture at different domains are analyzed.

(1) Predicted tensile nonlinear curves and matrix fragmentation density evolution curves agree with experimental data, and the matrix fragmentation approaches saturation before tensile fracture, and the interface partial debonding remains till tensile fracture.
(2) Microdamage parameters of first matrix fragmentation stress, saturation matrix fragmentation stress and density, composite tensile strength and failure strain are obtained from tensile stress–strain curves and can be used to characterize tensile nonlinear behavior of mini-CMCs.
(3) At higher fiber volume, the debonding fraction at the same applied stress decreases, and the composite strain at nonlinear Domain II decreases, and the composite tensile strength and failure strain increase.
(4) At higher interface shear stress and interface debonding energy, the debonding fraction at the same applied stress decreases, and the composite strain at nonlinear Domain II decreases, and the failure strain of the composite decreases.

## Figures and Tables

**Table 1:** Material properties of unidirectional SiC/SiC and C/SiC minicomposite.

Items	Hi-Nicalon^TM^ SiC/SiC [13]	Hi-Nicalon^TM^ Type S SiC/SiC [13]	Tyranno^TM^ ZMI SiC/SiC [13]	SiC/SiC [18]	T300^TM^ C/SiC [14]
*r*_f_/(μm)	7	6	5.5	6.5	3.5
*V*_f_/(%)	25.8	22.8	27.5	23	30
*E*_f_/(GPa)	270	400	170	122	120
*E*_m_/(GPa)	350	350	350	303	150
α_f_/(10^−6^/°C)	3.5	4.5	4.0	3.1	−0.38
α_m_/(10^−6^/°C)	4.6	4.6	4.6	4.6	2.8
*σ*_R_/(MPa)	420	350	280	220	180
*m*	6	6	8	3	5
*l*_sat_/(μm)	564	667	667	780	400
*m*_f_	5	5	5	5	5
*σ*_uts_/(MPa)	644	488	498	399	348
*ε*_f_/(%)	0.45	0.31	0.36	0.88	0.67
